# Performance verification of the new UF-1500 urine particle analyser: a new opportunity for small and medium laboratories

**DOI:** 10.1016/j.plabm.2025.e00481

**Published:** 2025-05-31

**Authors:** Giulia Previtali, Michela Seghezzi, Roberto Marozzi, Monica Fortino, Gianluca Agnolet, Mauro Barretta, Claudia Bizzoni, Valeria Bolla, Greta Bolzoni, Alessia Cesani, Matteo Diambrini, Sara Apassiti Esposito, Giorgia Giuliani, Alina Picciau, Maria Grazia Alessio

**Affiliations:** Clinical Chemistry Laboratory, Department of Laboratory Medicine, Papa Giovanni XXIII Hospital, Bergamo, Italy

**Keywords:** UF-1500, Automated urine analysis, Fuchs Rosenthal chamber, Urine particles, Analytical performance

## Abstract

**Background:**

The UF-1500 is the new fully automated urine particle analyser by Sysmex specifically tailored for small and medium laboratories. This study aim to validate its analytical and diagnostic performance.

**Methods:**

754 first morning mid-stream urines were analysed on UF-1500; 550 samples were used for the UF-5000 comparison and 204 were used for the correlation with manual count on Fuchs-Rosenthal chamber. Carry-over, linearity and imprecision of the UF-1500 were also assessed.

**Results:**

Correlation with the UF-5000 was excellent, with *r* coefficient range between 0.88 and 1.00. Correlation with Fuchs-Rosenthal chamber was very good for all the parameters; *r coefficient* ranged between 0.67 and 0.94. Linearity regression coefficient of determination (R^2^) was excellent for almost all the parameters. No carry-over was observed. The within-run imprecision range between 2.93 % for RBC and 35.63 % for WBC. The between-run imprecision ranged between 2.1 % for RBC and 23.9 % for CAST, using low and high positive quality controls, respectively.

**Conclusion:**

The analytical and diagnostic performance is satisfactory for almost all the parameters, when compared with the UF-5000; the correlation with the reference method is equally good.

## Highlights

• Automated analysis of urine particles.
• Verification of UF-1500 performance.
• Comparison of UF-1500 with UF-5000 and Fuchs Rosenthal chamber count.

## 1 Introduction

Urinalysis is the most commonly requested laboratory test to screen and follow up patients for diseases of the kidney and urinary tract due to metabolic or systemic diseases [1].

The analysis of sediment particles by manual microscopy evaluation is still considered as the gold standard method but it has some limitations: it is a time-consuming, labour-intensive procedure, with high imprecision and inaccuracy, it requires expert knowledge, and it is strongly influenced by the inter-observer variability [[2], [3], [4]].

The availability of automated analysers of urinary sediment has improved efficiency, standardization and accuracy of urinary sediment analysis [[4], [5], [6], [7], [8], [9], [10], [11], [12], [13], [14]]; particularly, it made urinalysis quicker and less observer dependent; in addition, the increased number of counted particles has improved the analytical precision [11]. These aspects are particularly useful when applied to small laboratories, where the skills of the operators may be restricted, due to the limited case mix. The UF-1500 is the new fully automated urine particle analyser of the Sysmex (Sysmex Corporation, Kobe, Japan) family: it is equipped with the same measurement principle as the UF-4000/UF-5000 but specifically projected for laboratories in the low and medium segment to offer a compact alternative of UF-4000/UF-5000 but with the same performances and parameters. It is based on the fluorescence flow cytometry method to recognize and classify 17 types of urinary particles: Red blood cells (RBC); Non lysed RBC (NLRBC); White Blood Cells (WBC); WBC clumps (WBCc); Total Epithelial Cells (EC); Non-Squamous Epithelial Cells (Non-SEC); Squamous Epithelial Cells (SEC); Transitional Epithelial Cells (Tran.EC); Renal Tubular Epithelial Cells (RTEC); CAST (CAST); Hyaline CAST (Hy.CAST); Pathological CAST (Path.CAST); Bacteria (BACT); Crystals (X'TAL); Yeast-like cells (YLC); Spermatozoa (SPERM) and Mucus (MUCUS). The objective of this study is to evaluate the performance of the UF-1500 analyser in a clinical laboratory setting using normal and abnormal urine specimens and to demonstrate that the UF-1500 has analytical performance similar to the UF-5000, and therefore it can help to improve the quality of urinalysis in small to medium size laboratories.

## 2 Material and methods

### 2.1 UF-1500 analyser technical details

As with the previous analysers of the UF-Series (UF-4000/UF-5000), the UF-1500 is equipped with a blue semiconductor laser (488 nm). By detecting forward scatter light (FSC), side scatter light (SSC), side fluorescent light (SFL) and depolarized side scattered light (DSS), it is able to recognize, count and classify the sediment components of urine.

The sample volume required for analysis is 2 mL (0.6 mL for STAT mode) with an aspiration volume of 0.68 mL (0.52 mL for STAT mode). The entire sequence of operations from sample aspiration to output of analysis results can be processed automatically. Analysis results are displayed on a touch panel, and can be output to a host computer. Quality control (high and low level) is a suspension of control particles representing RBC, WBC, EC, CAST and BACT in a specific buffer.

### 2.2 Sample selection

Between June and December 2022, 754 fresh, first morning, clean catch mid-stream voided urine samples were selected from those obtained for diagnostic urinalysis at the Clinical Chemistry Laboratory of the Papa Giovanni XXIII Hospital in Bergamo (Italy). The Papa Giovanni XXIII Hospital in Bergamo is a major public healthcare institution in Italy's Lombardy region. It offers a wide range of medical services, including emergency care, surgery, and diagnostics. Its laboratory diagnostics department is a key component, conducting over 5 million tests annually. Around 60–65 % of these tests (approximately 3 million) are for inpatients, aiding diagnosis, treatment, and recovery monitoring. The remaining tests support outpatient services, covering routine check-ups, chronic disease monitoring, pre-surgical screenings, and specialized diagnostics for conditions like diabetes, cancer, cardiovascular diseases, and infections. After the diagnostic analysis, the leftover samples were anonymized. Samples were selected with no exclusion criteria to try to cover the entire linearity range for all parameters and to create a mix of patients typical of routine sample series. When the required volume of a sample could not be obtained from a single patient, “minipools” by mixing samples from two patients were performed. To obtain urine samples with sperm, spermatic fluid was added to a negative for sperm urine sample; while first stream samples from patients from the Gynaecology Units were collected to obtain a high concentration of SEC. Bacteria was not considered in the present study for UF-1500 vs FRC comparison. Samples with insufficient volume, collected in vacuum tubes or with preservatives were excluded.

The study was approved by the local ethics committee and conducted in accordance with the Declaration of Helsinki.

### 2.3 UF-1500 vs UF-5000 comparison

550 samples were analysed two times on the UF-1500 (Sysmex Corporation, Kobe, Japan) and once on the UF-5000 (Sysmex Corporation, Kobe, Japan) after routine analysis and within 4 h after collection.

Two levels of Positive controls were performed at the beginning of each analytical session on both the UF-1500 and the UF-5000.

Passing-Bablok regression analysis, Pearson correlation and Bland-Altman bias plot analysis were evaluated for UF-1500 vs UF-5000, for all the parameters. Slopes and intercepts of the Passing-Bablok regression and Bland-Altman bias were calculated within 95 % confidence interval (95 %CI).

### 2.4 UF-1500 vs Fuchs-Rosenthal chamber counting

204 samples were selected for UF-1500 vs Fuchs-Rosenthal chamber (FRC) comparison. FRC counting was executed according to guidelines [15,16] with a phase contrast microscope using an eyepiece set at 10X and a lens set at 40X, for a total of 400X, and with a lens set at 20X (200X total) for casts. Manual microscopy was performed on uncentrifuged samples blinded to the results from the UF-1500. For all the particles, except RBC, 3.2 μL volume was used for the count; for RBC, if 200 RBC are counted in a volume less than 1 μL the RBC concentration/μL can be calculated based on the count of 1 μL volume.

Results were compared with those obtained by two measurements for each sample on the UF-1500.

Passing-Bablok regression analysis, Pearson correlation and Bland-Altman bias plot analysis were evaluated for UF-1500 vs Fuchs Rosenthal chamber, for the main parameters (RBC, WBC, SEC). Slopes and intercepts of the Passing-Bablok regression and Bland-Altman bias were calculated within 95 % confidence interval (95 %CI).

Sensitivity (SE), specificity (SP), positive (LR+) and negative (LR-) likelihood ratio, positive (PPV) and negative (NPV) predictive value were calculated for the main parameters (RBC, WBC, SEC) from the comparison study data (UF-1500 vs FRC). Area under the curve (AUC) of ROC was calculated on the UF-1500 data for the same parameters.

### 2.5 Carry-over

Carry-over measurement was conducted for RBC, WBC and BACT by analysing three aliquots of a highly positive concentration urine sample for each parameters (H1, H2, H3) followed by three aliquots of a completely negative urine sample (less than 1/μL for each parameter; B1, B2, B3). These measurements were repeated two times with different high concentrations.

Carry-over ratio was calculated with the following formula [17]:

Carry-over ratio = (B1-B3)/(H3-B3)x100 (%)

### 2.6 Linearity

The linearity was calculated for all the parameters according to the Clinical and Laboratory Standards Institute (CLSI) EP6-A, 2003 [18] using seven sample dilution scheme (for RBC, NLRBC, WBC, EC, SEC, Non-SEC, RTEC, CAST, Hy.CAST, Path.CAST, BACT, X’TAL, YLC, SPERM) or five sample dilution scheme (for TranEC).

Aliquots were obtained by mixing a different proportion of physiological saline and a selected sample with a high cellularity. Each aliquot was repeated 3 times in a working day, and the mean was calculated. A graphic representation of data was generated between measured and theoretical values and the linear regression coefficient (R^2^) was calculated.

### 2.7 Imprecision

The within-run imprecision of the UF-1500 was calculated according to the CLSI guidelines EP5-A [19]. Two urine samples with low and high concentration was selected for RBC, WBC and EC. Moreover, a sample with particles concentration near the clinically relevant cut off level was selected: 10 particles/μL for RBC, 20 particles/μL for WBC, 20 particles/μL for EC. Samples were repeated 40 times for each parameter and for each level.

The between-run imprecision was obtained for the same parameters by analysing the UF- Control high and low levels (Sysmex Corporation, Kobe, Japan) for 20 days consecutively.

### 2.8 Statistical analysis

Statistical analysis was carried out using Analyse-it™ software, version 5.92 (Analyse-it Software Ltd, Leeds, UK).

## 3 Results

A total of 754 samples were collected and analysed in this study; 550 samples were compared with the UF-1500 and the UF-5000, while 204 samples were compared with the UF-1500 and FRC.

Included patients ranged in age from 0 to 97 years, with a mean age of 57 years (standard deviation: 20.5 years). The great majority of the samples were from outpatients (59.8 %), and 47.5 % of samples were from female patients. Prior to conducting statistical analyses, all comparisons and parameters were reviewed to identify any samples warranting exclusion due to analytical or pre-analytical considerations The number of the excluded samples was not significant for all the parameters. For the comparison UF-1500 vs FRC, six samples were excluded (only for RBC) for being flagged by UF-1500 as “interference” by yeast on the count. In the comparison between the UF-1500 and the UF-5000, only one sample (for BACT parameter) was excluded.

### 3.1 UF-1500 vs UF-5000 comparison

**Fig. 1:**
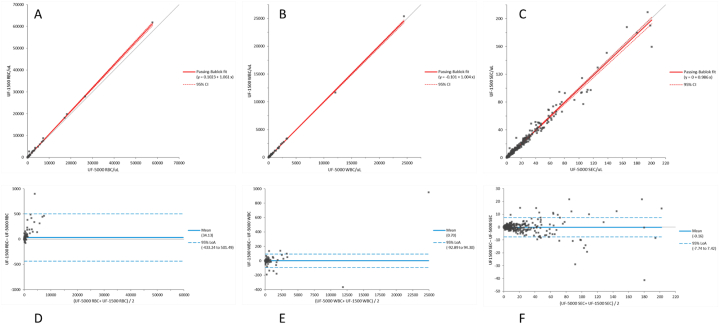
Comparison between the UF-1500 and Fuchs-Rosenthal chamber (FRC) count using Passing-Bablok regression and Bland-Altman Plot analysis. A. Passing–Bablok regression analysis for Red Blood Cells count by UF-1500 (UF-1500 RBC) with respect to the ones by FCR (FCR RBC): y = 1.03x+1.45 B. Passing–Bablok regression analysis for White Blood Cells count by UF-1500 (UF-1500 WBC) with respect to the ones by FCR (FCR WBC): y = 0.99x-0.02 C. Passing–Bablok regression analysis for Squamous Epithelial Cells count by UF-1500 (UF-1500 SEC) with respect to the ones by FCR (FCR SEC): y = 1.15x+0.03 D. Bland-Altman Plot analysis analysis for Red Blood Cells count by UF-1500 (UF-1500 RBC) with respect to the ones by FCR (FCR RBC): 15.1/μL (95 %CI: 71.1 to 40.9) E. Bland-Altman Plot analysis analysis for White Blood Cells count by UF-1500 (UF-1500 WBC) with respect to the ones by FCR (FCR WBC): 0.9/μL (95 %CI: 19.7 to 21.6) F. Bland-Altman Plot analysis for Squamous Epithelial Cells count by UF-1500 (UF-1500 SEC) with respect to the ones by FCR (FCR SEC): 0.75/μL (95 %CI: 1.1 to 2.6).

Comparison between the UF-1500 and the UF-5000 counts for all the parameters are summarized in Table 1. The Pearson's correlation showed a coefficient always up to 0.88 with p < 0.0001. Passing-Bablok regression showed slopes ranged between 0.71 (95 %CI, 0.65 to 0.79) for Hy.CAST parameter and 5.35 (95 %CI, 4.03 to 8.53) for Path.CAST parameter, the intercepts were comprised between −0.25 (95 %CI −0.94 to 0.24) for BACT count parameter and 0.10 (95 %CI −0.04 to 0.27) for RBC count parameters. The Bland–Altman analysis showed a significant positive bias (i.e., overestimation) for RBC, NLRBC and X’TAL parameters; while it showed a significant negative bias (i.e., underestimation) for Hy.CAST and CAST parameters. Fig. 1 showed the Passing-Bablok regression and Bland-Altman plot analysis of the principal parameters*.* Moreover, we conducted a comparability analysis of the two methods as a qualitative comparison using defined cutoffs. The results are summarized in Table 2. These comparisons showed good results, with total agreement ranging between 0.94 (for RBC) and 0.99 (for WBC), and Cohen's kappa ranging between 0.88 (for RBC) and 0.97 (for WBC).

**Table 1:** Comparison of the UF-5000 with the UF-1500 for all parameters.

Parameters	Median value (range)(/μL)	Pearson (r value)	Passing Bablok regression		Bland Altman bias (μL) (95 %CI)
			Slope (95 %CI)	Intercept (95 %CI)	
**RBC** (550 samples)	UF-5000 = 11.0 (0–57682) UF-1500 = 11.6 (0–61830)	1.00	1.06 (1.04–1.07)	0.10 (−0.04 to 0.27)	34.1 (14.2–54.1)
**NLRBC** (550 samples)	UF-5000 = 10 (0–57607) UF-1500 = 10.4 (0–61698)	0.99	1.06 (1.04–1.07)	0.06 (−0.05 to 0.15)	34.55 (14.66–54.43)
**WBC** (550 samples)	UF-5000 = 13.9 (0-24443 UF-1500 = 13.2 (0–25395)	1.00	1.00 (0.99–1.01)	−0.10 (−0.23 to 0.00)	0.7 (−3.3 to 4.7)
**WBCc** (550 samples)	UF-5000 = 0 (0–1430) UF-1500 = 0 (0–1426)	0.99	0.92 (0.87–0.97)	0 (0–0)	−0.12 (−0.73 to 0.49)
**EC** (550 samples)	UF-5000 = 6.9 (0–219) UF-1500 = 7.3 (0–233)	0.99	1.00 (0.98–1.02)	−0.05 (−0.26 to 0.05)	−0.11 (−0.49 to 0.26)
**SEC** (550 samples)	UF-5000 = 3.0 (0–200.8) UF-1500 = 2.9 (0–209.5)	0.99	0.99 (0.96–1.00)	0 (−0.05 to 0.02)	−0.16 (−0.49 to 0.16)
**Non-SEC** (550 samples)	UF-5000 = 2.4 (0–51.1) UF-1500 = 2.3 (0–50.7)	0.96	1.00 (0.98–1.03)	0 (−0.01 to 0.05)	0.05 (−0.10 to 0.19)
**RTEC** (550 samples)	UF-5000 = 2.1 (0–50.4) UF-1500 = 2.0 (0–49.7)	0.96	1.00 (0.99–1.03)	0 (0–0)	0.05 (−0.09 to 0.19)
**Tran.EC** (550 samples)	UF-5000 = 0.1 (0–10.3) UF-1500 = 0.1 (0–14.6)	0.92	0.98 (0.86–1.00)	0 (0–0)	−0.01 (−0.04 to 0.03)
**CAST** (550 samples)	UF-5000 = 0.2 (0–16.7) UF-1500 = 0.2 (0–18.4)	0.93	0.78 (0.71–0.87)	0 (0–0)	−0.18 (−024 to −0.12)
**Path.CAST** (550 samples)	UF-5000 = 0.0 (0–7.8) UF-1500 = 0.0 (0–16.5)	0.95	5.35 (4.03–8.53)	0 (0–0)	−0.02 (−0.04 to 0.0005)
**Hy.CAST** (550 samples)	UF-5000 = 0.1 (0–14.1) UF-1500 = 0.1 (0.2–16.5)	0.88	0.71 (0.65–0.79)	0 (0–0)	−0.16 (−0.22 to −0.09)
**SPERM** (550 samples)	UF-5000 = 0 (0–723) UF-1500 = 0 (0–777)	0.99	1.04 (0.95–1.07)	0 (0–0)	0.01 (−042 to 0.44)
**YLC** (550 samples)	UF-5000 = 0.3 (0–3546) UF-1500 = 0.4 (0–4178)	0.98	1.11 (1.04–1.20)	0.03 (0.00 o 0.05)	2.37 (−1.93 to 6.67)
**X’TAL** (550 samples)	UF-5000 = 0.3 (0–615) UF-1500 = 0.9 (0–894)	0.99	1.44 (1.32–1.66)	0.30 (0.23–0.35)	2.40 (1.28–3.53)
**BACT** (549 samples)	UF-5000 = 81.2 (0–100000) UF-1500 = 64.6 (0–100000)	0.97	0.99 (0.98–1.00)	−0.25 (−0.94 to 0.24)	10.34 (−420.4 to 441.11)

Red blood cells (RBC); Non lysed RBC (NLRBC); White Blood Cells (WBC); WBC clumps (WBCc); Hyaline Casts (Hy.CAST); Pathologycal Casts (Path.CAST); Total Epithelial Cells (EC); Squamous Epithelial Cells (SEC); Non-Squamous Epithelial Cells (Non-SEC); Transitional Epithelial Cells (Tran.EC); Renal Tubular Epithelial Cells (RTEC); Yeast-like cells (YLC); Spermatozoa (SPERM); Crystals (X'TAL).

**Table 2:** Agreement between the UF-5000 and the UF-1500 for the main parameters.

Parameter	Positive Agreement	Negative Agreement	Total Agreement	Cohen's kappa
**RBC** (cut-off: >10/μL)	0.93 (274/288)	0.95 (244/262)	0.94	0.88
**WBC** (cut-off: >20/μL)	0.98 (231/234)	0.99 (311/316)	0.99	0.97
**EC** (cut-off: >20/μL)	0.94 (133/136)	0.99 (406/414)	0.98	0.95
**BACT** (cut-off: >500/μL)	0.96 (140/150)	0.98 (394/400)	0.97	0.93

Red blood cells (RBC); White Blood Cells (WBC); Bacteria (BACT).

### 3.2 Comparison with the reference method (FRC) and accuracy performance

**Fig. 2:**
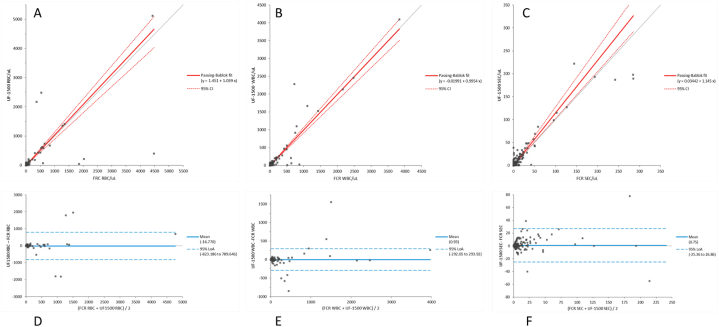
Comparison between the UF-1500 and Fuchs-Rosenthal chamber (FRC) count using Passing-Bablok regression and Bland-Altman Plot analysis. A. Passing–Bablok regression analysis for Red Blood Cells count by UF-1500 (UF-1500 RBC) with respect to the ones by FCR (FCR RBC): y = 1.03x+1.45 B. Passing–Bablok regression analysis for White Blood Cells count by UF-1500 (UF-1500 WBC) with respect to the ones by FCR (FCR WBC): y = 0.99x-0.02 C. Passing–Bablok regression analysis for Squamous Epithelial Cells count by UF-1500 (UF-1500 SEC) with respect to the ones by FCR (FCR SEC): y = 1.15x+0.03 D. Bland-Altman Plot analysis analysis for Red Blood Cells count by UF-1500 (UF-1500 RBC) with respect to the ones by FCR (FCR RBC): 15.1/μL (95 %CI: 71.1 to 40.9) E. Bland-Altman Plot analysis analysis for White Blood Cells count by UF-1500 (UF-1500 WBC) with respect to the ones by FCR (FCR WBC): 0.9/μL (95 %CI: 19.7 to 21.6) F. Bland-Altman Plot analysis for Squamous Epithelial Cells count by UF-1500 (UF-1500 SEC) with respect to the ones by FCR (FCR SEC): 0.75/μL (95 %CI: 1.1 to 2.6).

Comparison between the UF-1500 and the reference method (FRC) for RBC, WBC and SEC are summarized in Fig. 2. The comparison analysis was not evaluated for Tran.EC, RTEC and YLC because of the limited number of positive samples (<50) collected in our study. Passing-Bablok regression slope ranged from 0.99 (95 %CI, 0.91 to 1.07) for WBC to 1.15 (95 %CI, 1.02 to 1.28) for SEC and intercept ranged from −0.02 (95 %CI, −0.38 to 0.26) for WBC to 1.45 (95 %CI, 1.11 to 2.5) for RBC. The Bland Altman Bias was −15.1/μL (95 %CI, −71.1 to 40.9) for RBC, 0.9/μL (95 %CI, −19.7 to 21.6) for WBC and 0.8/μL (95 %CI, −1.1 to 2.6) for SEC. The mean bias calculated in all data range was not statistically significant for all the three parameters. Considering only samples with particles concentration ranging from 1 to 20/μL, near to clinical decision levels, the mean bias observed was even significantly better for the three parameters (data not showed). Using Pearson statistics, the correlation factor between the UF-1500 and FRC counting showed *r* > 0.90 for all the three parameters. The diagnostic performance of the analyser was evaluated through the ROC analysis for the three main parameters (RBC, WBC, SEC) and the area under the curve (AUC) obtained was very good (>0.85). Sensitivity, specificity, likelihood ratios and predictive values are shown in Table 3: the negative predictive values obtained for the three parameters is high, suitable for a screening technique.

**Table 3:** Diagnostic accuracy performance of the UF-1500 for the most frequent parameters.

	FRC			UF-1500												
	Cut Off	POS	NEG	Cut Off	AUC	TP	FP	TN	FN	SE	SP	PP	LR +	LR-	PPV	NPV
**RBC**	10.00	82	115	10.05	0.85	74	47	68	8	0.84	0.71	0.42	2.93	0.22	0.68	0.86
**WBC**	10.00	107	97	10.0	0.98	98	0	97	9	0.92	1.00	0.52	+∞	0.08	1.00	0.92
**SEC**	10.00	47	157	10.9	0.90	39	12	145	8	0.83	0.92	0.23	10.86	0.18	0.76	0.95

Red blood cells (RBC); White Blood Cells (WBC); Squamous Epithelial Cells (SEC); Fuchs-Rosenthal chamber (FRC); Area Under the Curve (AUC); True Positive (TP); False Positive (FP); True Negative (TN); False Negative(FN); Sensitivity (SE); Specificity (SP); Prior Probability (PP); Positive Likelihood Ratio (LR+); Negative Likelihood Ratio (LR-); Positive Predictive Value (PPV); Negative Predictive Value (NPV).

### 3.3 Carry-over

The carry-over of the UF-1500 was evaluated with the specific formula on the parameters that are likely to be present in high concentration in the urine samples (RBC, WBC, BACT) and it was found to be excellent for all the considered parameters, with complete absence of sample carry-over (<0.05 %). Results are shown in Table 4.

**Table 4:** Carry-over performance the UF-1500.

	High value (mean/μL)	Low value (mean/μL)	Carryover results (%)	Target (% or/μL)
**RBC**	Sample 1: 14660 Sample 2: 19222.4	Sample 1: 5.2 Sample 2: 3.1	Sample 1: 0 Sample 2: 0	0.05 % or less 10.0/μL or less
**WBC**	Sample 1: 854.9 Sample 2: 4725.6	Sample 1: 2.6 Sample 2: 2.4	Sample 1: 0 Sample 2: 0	0.05 % or less 10.0/μL or less
**BACT**	Sample 1: 39351.7 Sample 2:14967.5	Sample 1: 1.2 Sample 2: 2.0	Sample 1: 0 Sample 2: 0	0.05 % or less 5.0/μL or less

Red blood cells (RBC); White Blood Cells (WBC); Bacteria (BACT).

### 3.4 Linearity

Linearity was conducted in a wide range of value for all the principal parameters and linear regression showed to be the best fitting model for all the parameters. The linear regression coefficient of determination (R^2^) for all parameters was very good for all the parameters (R^2^ > 0.950). Overall, all parameters reached an excellent regression (R^2^ > 0.990) except for Tran.EC and SPERM parameters. Results are shown in Table 5.

**Table 5:** Linearity performance the UF-1500.

Parameters	Range [/μL]	Coefficient of determination(R^2^)	Linearity criteria according to manufacture’ specifications
**RBC**	1.1 to 22616.4	1.000	0.950 or more
**NLRBC**	1.9 to 22369.4	0.999	0.950 or more
**WBC**	1.0 to 22906.5	0.996	0.950 or more
**EC**	0.4 to 256.4	0.999	0.950 or more
**SEC**	0.2 to 238.8	0.999	0.950 or more
**Non-SEC**	0.7 to 89.5	0.992	0.950 or more
**RTEC**	0.5 to 87.9	0.992	0.950 or more
**Tran.EC**	0.0 to 24.6	0.956	0.950 or more
**CAST**	0.00 to 28.88	0.989	0.950 or more
**Path.CAST**	0.00 to 22.67	0.988	0.950 or more
**Hy.CAST**	0.00 to 30.36	0.995	0.950 or more
**SPERM**	0.0 to 433.7	0.975	0.950 or more
**YLC**	0.2 to 2391.5	0.996	0.950 or more
**X’TAL**	4.8 to 235.1	0.997	0.950 or more
**BACT**	4.0 to 11715.9	0.996	0.950 or more

Red blood cells (RBC); Non lysed RBC (NLRBC); White Blood Cells (WBC); Total Epithelial Cells (EC); Squamous Epithelial Cells (SEC); Non-Squamous Epithelial Cells (Non-SEC); Transitional Epithelial Cells (Tran.EC); Renal Tubular Epithelial Cells (RTEC); Hyaline Casts (Hy.CAST); Pathologycal Casts (Path.CAST); Bacteria (BACT); Crystals (X'TAL); Yeast-like cells (YLC); Spermatozoa (SPERM).

### 3.5 Imprecision

All the three main parameters (RBC, WBC and EC) showed an acceptable coefficient of variation (CV%) on the three series. In particular, the imprecision was 26.6 %, 19.9 %, 2.9 % for a mean concentration of 3.0/μL, 11.2/μL, 7585.8/μL for RBC; 35.6 %, 7.1 %, 4.2 % for a mean concentration of 1.3/μL, 38.8/μL, 3659.6/μL for WBC and 31.3 %, 12.9 %, 11.8 % for a mean concentration of 2.7/μL, 30.7/μL, 119.8/μL for EC, respectively.

The between-run imprecision was calculated using the data obtained in 20 days consecutively, from two levels of quality control samples performed at the beginning of every analytical session. CV% ranged between 2.1 % for RBC at the concentration of 231.5/μL and 23.9 % for CAST at the concentration of 3.5/μL.

CV was 6.5 % and 2.1 % for RBC at the mean concentration of 40.8/μL and 231.5/μL; 14.2 % and 3.5 % for WBC at the mean concentration of 41.3/μL and 887.6/μL; 21.9 % and 10.4 % for EC at the mean concentration of 9.6/μL and 79.6/μL; 23.9 % and 17.2 % for CAST at the mean concentration of 3.5/μL and 17.7/μL; 9.4 % and 4.8 % for BACT at the mean concentration of 208.7/μL and 892.9/μL respectively.

## 4 Discussion

During the last decades, the widespread of automated analyzers improved urinalysis standardization and reduced inter-observer variability due to operator skills; analysis imprecision was improved thanks to the ability of the analyzers to count even great number of particles and the efficiency of the laboratory process advanced along with the identification of various type of urinary elements in the last generation of analyzers [8,14,20].

These reasons led to the publication of Italian Guidelines for the analytical phase of urinalysis in 2016 by Intersociety Urinalysis Group that recommended the implementation of automated systems for urinary sediment also for laboratories that analyse 50–100 urine samples per day [21].

In Italy, the reality of laboratories is represented by mainly small and medium-sized laboratories, especially in the southern part of the country.

Recently, the Centre for the Quality of the Laboratories of the Lombardy Region, one of the most populated and economically advanced areas, has implemented a complete External Quality Assessment (EQA) on urinalysis for all public and private laboratories. Among others, it includes images of several particles of the urinary sediment and each laboratories must recognize and correlate them to a specific clinical condition. Analysis of the results showed that more than 30 % of the laboratories perform urinalysis without any type of automation, and this percentage is certainly higher in other areas of the country. 5 % of laboratories perform urinalysis only by dipstick without the sediment evaluation and in 26,7 % the sediment evaluation is performed exclusively by manual microscopy count. This analysis also showed that a significant percentage of laboratories are unable to correctly identify some of the main elements of the sediment, including deep transitional cells, tubular cells, and casts. These are mainly small laboratories, without automation support, where the operators have low skills for the recognition of urinary particles, also due to a limited case mix of samples. This situation causes an objective difficulty to recognize the rarest elements of urine samples. Therefore, it is of great importance to have automated urinary sediment analysers designed for small numbers of urine to improve standardization and quality. To our knowledge, nowadays, there are no instruments designed specifically for small and medium size laboratory. The new UF -1500 can be the solution for this requirement.

A paper published by Manoni et al. [10] in 2010 already showed that the UF-1000i was an efficient alternative to the manual microscopy, which has well-known limitations [22]. Later in 2017, Previtali et al. evaluated the performance of that last generation cytometry the UF-5000 by comparison with the reference method (manual microscopy with FRC counting), confirming that it is able to recognize most of the particles of the urinary sediment with efficiency equal to or higher than the main competitors, especially for the elements with greater clinical impact, such as casts and tubular cells. [11]. This finding was confirmed in other recent publications, regarding the diagnosis of upper urinary tract infection and differentiation of the origin of microhaematuria [12,13,23,24].

The comparison study between the UF-1500 and the UF-5000 on 550 fresh first morning, clean catch mid-stream urine samples showed that analytical and diagnostic performance of the new analyser is satisfactory for almost all the parameters, with results similar to the UF-5000. The results showed in Table 1demonstrate that the UF-1500 can ensure high reliability performance.

Using samples with a wide range of values for all the parameters is essential for an optimal evaluation, because performance can be significantly affected by different concentrations, especially the higher ones. In this paper, we compared samples with a wide range of element's concentration, as showed in Table 1. In recent paper that evaluated automated urine analyzers, the range of values used was very limited [7,[25], [26], [27], [28]] or not declared [2]; thus, it is difficult to estimate the performance of these instruments with high concentrated samples, a common finding in the laboratory routine.

The second protocol of this study was the comparison between the UF-1500 and FCR counting results on 204 urine samples, with wide range of values for each parameters, as described by international guidelines [15,29,30]. FRC count was carried by a pathologist with proven experience on the evaluation of urinary sediment, blinded to the results of the UF-1500; for each samples, 3.2 μL were counted, and 1 μL for samples with more than 200 RBC/μL. Comparison data was very good for the three main parameters (RBC, WBC and EC) by applying Pearson statistics, Passing-Bablock regression and Bland-Altman bias analysis. A good correlation was found also for SPERMS, YLC and X’TAL (data not showed). The comparison analysis was not evaluated for Tran.EC, RTEC and YLC because of the limited number of positive samples collected. These data suggest that the UF-1500, as the previous the UF-5000, provides accurate counts in comparison with the microscopic counting.

In our study, no carry-over was found for the considered parameters (RBC, WBC, BACT), with an increment of the counts <0.05 %. These results proving that the washing system of the UF-1500 is efficient.

The linearity evaluation of the UF-1500 demonstrated excellent and clinically significant performance for all parameters (R^2^ > 0.990), with the exception of Transitional Epithelial Cells (Tran. EC) and Sperm. Within-run imprecision was calculated on fresh samples while between-run imprecision was performed using the control materials on two levels (low and high). Both evaluations showed very good results for all the parameters considered, similar or better to those obtained by other authors on similar analysers [11,13,25].

The comparison of our results with similar evaluation on different analyzers is difficult. A very low number of paper used FRC counting on uncentrifuged urine samples to evaluate the diagnostic performances of automated analyzers, due to the need of high skilled operators and the time-consuming of this procedure. [15,29,30]. Some authors used Kova slides or similar [25,26] or glass slide with coverslip and a fixed volume and/or centrifuged sample [1,2,7,27] and, in some cases, bright field microscope, instead of contrast phase microscope, was used [1]. Reference values for every parameters are different and technology-dependent; even samples’ selection (paediatric vs adult; healthy vs pathological; inpatients vs outpatients etc) can be a further element of variability.

Overall, our evaluation of the UF-1500 is positive; the analytical and diagnostic performance is satisfactory for almost all the parameters, when compared with the previous analyzers of the UF-Series (UF-4000/UF-5000), and the correlation with the reference method (microscopic evaluation on FRC) is equally good. The UF-1500 is as a reliable backup and fill in the same the UF-5000 performance specification for urinalysis mode. Despite a small number of samples analysed, this work remains one of the few in which this method of comparison is used, as suggested by the main guidelines.

In conclusion, the UF-1500 meets quality standards and high analytical performance as well as the UF-5000. For these reasons, small to medium size laboratories are now able to improve urinalysis automation with a sophisticated urine particle analyser, leading to an increase of standardization and quality of the urinalysis.

## CRediT authorship contribution statement

**Giulia Previtali:** Writing – review & editing, Writing – original draft, Methodology, Investigation, Conceptualization. **Michela Seghezzi:** Writing – review & editing, Writing – original draft, Methodology, Investigation, Conceptualization. **Roberto Marozzi:** Writing – review & editing, Software, Formal analysis. **Monica Fortino:** Methodology, Investigation, Data curation. **Gianluca Agnolet:** Methodology, Investigation, Data curation. **Mauro Barretta:** Methodology, Investigation, Data curation. **Claudia Bizzoni:** Methodology, Investigation, Data curation. **Valeria Bolla:** Methodology, Investigation, Data curation. **Greta Bolzoni:** Methodology, Investigation, Data curation. **Alessia Cesani:** Methodology, Investigation, Data curation. **Matteo Diambrini:** Methodology, Investigation, Data curation. **Sara Apassiti Esposito:** Methodology, Investigation, Data curation. **Giorgia Giuliani:** Methodology, Investigation, Data curation. **Alina Picciau:** Methodology, Investigation, Data curation. **Maria Grazia Alessio:** Writing – review & editing, Writing – original draft, Supervision, Project administration.

## Data availability statement

The data supporting this study's findings are available from the corresponding author upon reasonable request.

## Funding

The study was financially supported by Sysmex Europe GmbH.

## Declaration of competing interest

The authors declare that they have no competing interest or personal relationships that could have appeared to influence the work reported in this paper.

## Data Availability

Data will be made available on request.

## References

[1] Kucukgergin C., Ademoglu E., Omer B., Genc S. (2019). Performance of automated urine analyzers using flow cytometric and digital image-based technology in routine analysis. Scand. J. Clin. Lab. Invest..

[2] Liu H., Li Q., Zhang Y., Huang D., Yu F. (2022). Consistency analysis of the Sysmex UF-5000 and Atellica UAS 800 urine sedimentation analyzers. J. Clin. Lab. Anal..

[3] Winkel P., Statland B.E., Jorgensen K. (1974). Urine microscopy: an ill defined method examined by a multifactorial technique. Clin. Chem..

[4] Gadeholt H. (1964). Quantitative estimation of urinary sediment with special regard to sources of error. Br. Med. J..

[5] Delanghe J.R., Kouri T.T., Huber A.R., Hannemann-Pohl K., Guder W.G., Lun A., Sinha P., Stamminger G., Beier L. (2000). The role of automated particle flow cytometry in clinical practice. Clin. Chim. Acta.

[6] Becker G.J., Garigali G., Fogazzi G.B. (2016). Advances in urine microscopy. Am. J. Kidney Dis..

[7] Aper S.J.A., Gijzen K., Luimstra J.J., van der Valk J.T.M.H., Russcher A., Koçer R.G. (2021). Evaluation of the tellica® UAS 800: a new member of the automated urine sediment analyzer family. Scand. J. Clin. Lab. Invest..

[8] Delanghe J.R. (2007). New screening diagnostic techniques in urinalysis. Acta Clin. Belg..

[9] Hannemann-Pohl K., Kampf S.C. (1999). Automation of urine sediment examination: a comparison of the Sysmex UF-100 automated flow-cytometer with routine manual diagnosis (microscopy, test strips, and bacterial culture). Clin. Chem. Lab. Med..

[10] Manoni F., Tinello A., Fornasiero L., Hoffer P., Temporin V., Valverde S. (2010). G. Gessoni, Urine particle evaluation: a comparison between the UF-1000i and quantitative microscopy. Clin. Chem. Lab. Med..

[11] Previtali G., Ravasio R., Seghezzi M., Buoro S., Alessio M.G. (2017). Performance evaluation of the new fully automated urine particle analyser UF-5000 compared to the reference method of the Fuchs Rosenthal chamber. Clin. Chim. Acta.

[12] Kim H., Kim Y.O., Kim Y., Suh J.S., Cho E.J., Lee H.K. (2019). Small red blood cell fraction on the UF-1000i urine analyser as a screening tool to detect dysmorphic red blood cells for diagnosing glomerulonephritis. Ann. Lab.Med..

[13] Mizuno G., Hoshi M., Nakamoto K., Sakurai M., Nagashima K., Fujita T. (2021). Evaluation of red blood cell parameters provided by the UF-5000 urine auto analyser in patients with glomerulonephritis. Clin. Chem. Lab. Med..

[14] Oyaert M., Delanghe J. (2019). Progress in automated urinalysis. Ann. Lab.Med..

[15] Kouri T, Hofmann W, Falbo R, Oyaert M, Schubert S, Gertsen JB, et al,on behalf of the EFLM European Urinalysis Group. The EFLM European Urinalysis Guideline Update 2023 (EFLM Website). 10.1515/cclm-2024-0070 38534005

[16] Kouri T., Gyory A., Rowan R.M. (2003). ISLH Urinalysis Task Force. ISLH recommended reference procedure for the enumeration of particles in urine. Lab Haematol.

[17] Broughton P.M., Gowenlock A.H., McCormack J.J., Neill D.W. (1974). A revised scheme for evaluation of automatic instruments for use in clinical chemistry. Ann. Clin. Biochem..

[18] Clinical and Laboratory Standards Institute (2003).

[19] Clinical and Laboratory Standards Institute (1999).

[20] Oyaert M., Speeckaert M.M., Delanghe J.R. (2019). Estimated urinary osmolality based on combined urinalysis parameters: a critical evaluation. Clin. Chem. Lab. Med..

[21] Manoni F., Gessoni G., Fogazzi G.B., Alessio M.G., Caleffi A., Gambaro G. (2016). Physical, chemical and morphological urine examination guidelines for the analytical phase from the Intersociety Urinalysis Group, Italian. J. Ital. Nefrol.

[22] Sánchez-Mora C., Acevedo D., Porres M.A., Chaqués A.M., Zapardiel J., Gallego-Cabrera A. (2017). Comparison of automated devices UX-2000 and SediMAX/AutionMax for urine samples screening: a multicenter study. Clin. Biochem..

[23] Oyaert M., Speeckaert M., Boelens J., Delanghe J.R. (2020). Renal tubular epithelial cells add value in the diagnosis of upper urinary tract infection. Clin. Chem. Lab. Med..

[24] Hyodo T., Kumano K., Sakai T. (1999). Differential diagnosis between glomerular and nonglomerular haematuria by automated urinary flow cytometer. Nephron.

[25] Bogaert L., Peeters B., Billen J. (2017). Evaluation of a new automated microscopy urine sediment analyser- sediMAX conTRUST. Acta Clin. Belg..

[26] Falbo R., Sala M.R., Bussetti M., Cappellini F., Giacobone C., Fania C. (2019). Performance evaluation of a new and improved cuvette-based automated urinalysis analyzer with phase contrast microscopy. Clin. Chim. Acta.

[27] Nikler A., Čičak H., Bejuk D., Radišić Biljak V., Šimundić A.M. (2022). Verification of Atellica 1500 and comparison with Iris urine analyser and urine culture. Biochem. Med..

[28] Tantisaranon P., Dumkengkhachornwong K., Aiadsakun P., Hnoonual A. (2021). A comparison of automated urine analyzers cobas 6500, UN 3000-111b and iRICELL 3000 with manual microscopic urinalysis. Pract. Lab. Med..

[29] Cwiklińska A., Kąkol J., Kuchta A., Kortas-Stempak B., Pacanis A., Rogulski J. (2012). The standardization of urine particle counting in medical laboratories--a Polish experience with the EQA programme. Scand. J. Clin. Lab. Invest..

[30] Fogazzi G.B., Delanghe J. (2018). Microscopic examination of urine sediment: phase contrast versus bright field. Clin. Chim. Acta.

